# *RET* fusion genes in pediatric and adult thyroid carcinomas: cohort characteristics and prognosis

**DOI:** 10.1530/ERC-23-0117

**Published:** 2023-10-26

**Authors:** Barbora Bulanova Pekova, Vlasta Sykorova, Karolina Mastnikova, Eliska Vaclavikova, Jitka Moravcova, Petr Vlcek, Lucie Lancova, Petr Lastuvka, Rami Katra, Petr Bavor, Daniela Kodetova, Martin Chovanec, Jana Drozenova, Radoslav Matej, Jaromir Astl, Jiri Hlozek, Petr Hrabal, Josef Vcelak, Bela Bendlova

**Affiliations:** 1 Department of Molecular Endocrinology, Institute of Endocrinology, Prague, Czech Republic; 2 Department of Nuclear Medicine and Endocrinology, 2nd Faculty of Medicine, Charles University and Motol University Hospital, Prague, Czech Republic; 3 Departments of Otorhinolaryngology and Head and Neck Surgery, 1st Faculty of Medicine, Charles University and Motol University Hospital, Prague, Czech Republic; 4 Department of Ear, Nose and Throat, 2nd Faculty of Medicine, Charles University and Motol University Hospital, Prague, Czech Republic; 5 Department of Surgery, 2nd Faculty of Medicine, Charles University and Motol University Hospital, Prague, Czech Republic; 6 Department of Pathology and Molecular Medicine, 2nd Faculty of Medicine, Charles University and Motol University Hospital, Prague, Czech Republic; 7 Department of Otorhinolaryngology, 3rd Faculty of Medicine, University Hospital Kralovske Vinohrady, Prague, Czech Republic; 8 Department of Pathology, 3rd Faculty of Medicine, University Hospital Kralovske Vinohrady, Prague, Czech Republic; 9 Department of Otorhinolaryngology and Maxillofacial Surgery, 3rd Faculty of Medicine and Military University Hospital, Prague, Czech Republic; 10 Department of Pathology, Military University Hospital, Prague, Czech Republic

**Keywords:** *RET*, fusion gene, rearrangement, papillary thyroid carcinoma, outcome

## Abstract

Thyroid cancer is associated with a broad range of different mutations, including *RET* (rearranged during transfection) fusion genes. The importance of characterizing *RET* fusion-positive tumors has recently increased due to the possibility of targeted treatment. The aim of this study was to identify *RET* fusion-positive thyroid tumors, correlate them with clinicopathological features, compare them with other mutated carcinomas, and evaluate long-term follow-up of patients. The cohort consisted of 1564 different thyroid tissue samples (including 1164 thyroid carcinoma samples) from pediatric and adult patients. Samples were analyzed for known driver mutations occurring in thyroid cancer. Negative samples were subjected to extensive *RET* fusion gene analyses using next-generation sequencing and real-time PCR. *RET* fusion genes were not detected in any low-risk neoplasm or benign thyroid tissue and were detected only in papillary thyroid carcinomas (PTCs), in 113/993 (11.4%) patients, three times more frequently in pediatric and adolescent patients (29.8%) than in adult patients (8.7%). A total of 20 types of *RET* fusions were identified. *RET* fusion-positive carcinomas were associated with aggressive tumor behavior, including high rates of lymph node (75.2%) and distant metastases (18.6%), significantly higher than in *NTRK* fusion, *BRAF* V600E and *RAS*-positive carcinomas. Local and distant metastases were also frequently found in patients with microcarcinomas positive for the *RET* fusions. ’True recurrences’ occurred rarely (2.4%) and only in adult patients. The 2-, 5-, 10-year disease-specific survival rates were 99%, 96%, and 95%, respectively. *RET* fusion-positive carcinomas were associated with high invasiveness and metastatic activity, but probably due to intensive treatment with low patient mortality.

## Introduction

The incidence of thyroid cancer is increasing rapidly worldwide. Uncovering the genetic background of thyroid carcinomas can help determine the diagnosis, prognosis and appropriate treatment of patients. The broad spectrum of genetic alterations found in thyroid cancer includes different types of mutations: somatic point mutations, indels, copy number alterations, and fusion genes (Nikiforov 2011).

Among the fusion genes, the most common and best known are *RET* (rearranged during transfection) fusion genes (also known as *RET/PTC* rearrangements) arising from a chromosomal rearrangement between the *RET* gene and its partner gene. Expression of the fusion gene is driven by the promoter of the partner gene, leading to constitutive activation of the kinase domain of the *RET* gene. This leads to stimulation of the MAPK and PI3K pathways and oncogenesis (Arighi *et al.* 2005).

The *CCDC6/RET* (also known as *RET/PTC1*) fusion gene was discovered in 1987 and described 3 years later (Fusco *et al.* 1987, Grieco *et al.* 1990). It was one of the first reported genetic causes of thyroid cancer. A few years later, *PRKAR1A/RET* (*RET/PTC2*) and *NCOA4/RET* (*RET/PTC3*) fusions were identified (Bongarzone *et al.* 1993, Santoro *et al.* 1994). *RET* fusion genes have been studied in association with exposure to ionizing radiation during childhood and the development of thyroid cancer. The genetic landscape of radiation-induced papillary thyroid carcinomas (PTCs) was compared with sporadic PTCs in pediatric patients. *RET* fusions were highly prevalent in both pediatric PTC cohorts but occurred more frequently in radiation-induced PTCs with a predominance of *NCOA4/RET* fusion. In sporadic cases, *CCDC6/RET* fusion was the most common genetic alteration in pediatric thyroid cancer (Nikiforov *et al.* 1997, Ricarte-Filho *et al.* 2013). In adult cohorts, *RET* fusion genes were detected less frequently than in pediatric cohorts (Santoro *et al.* 2020). Studies presenting long-term follow-up of patients with *RET* fusion-positive carcinoma are almost absent. Interest in *RET* fusion genes has recently increased due to the novel possibility of targeted treatment with highly potent and selective RET inhibitors – selpercatinib and pralsetinib (Markham 2020*a*,*b*).

The objectives of this study were as follows: (i) analysis of genetic alterations with emphasis on *RET* fusion genes in a large series of local malignant and benign thyroid diseases, (ii) characterization of *RET* fusion-positive cases based on clinical and pathological data, and (iii) evaluation of long-term follow-up of patients with *RET* fusion-positive thyroid carcinoma.

## Materials and methods

### The cohort

**Figure 1:**
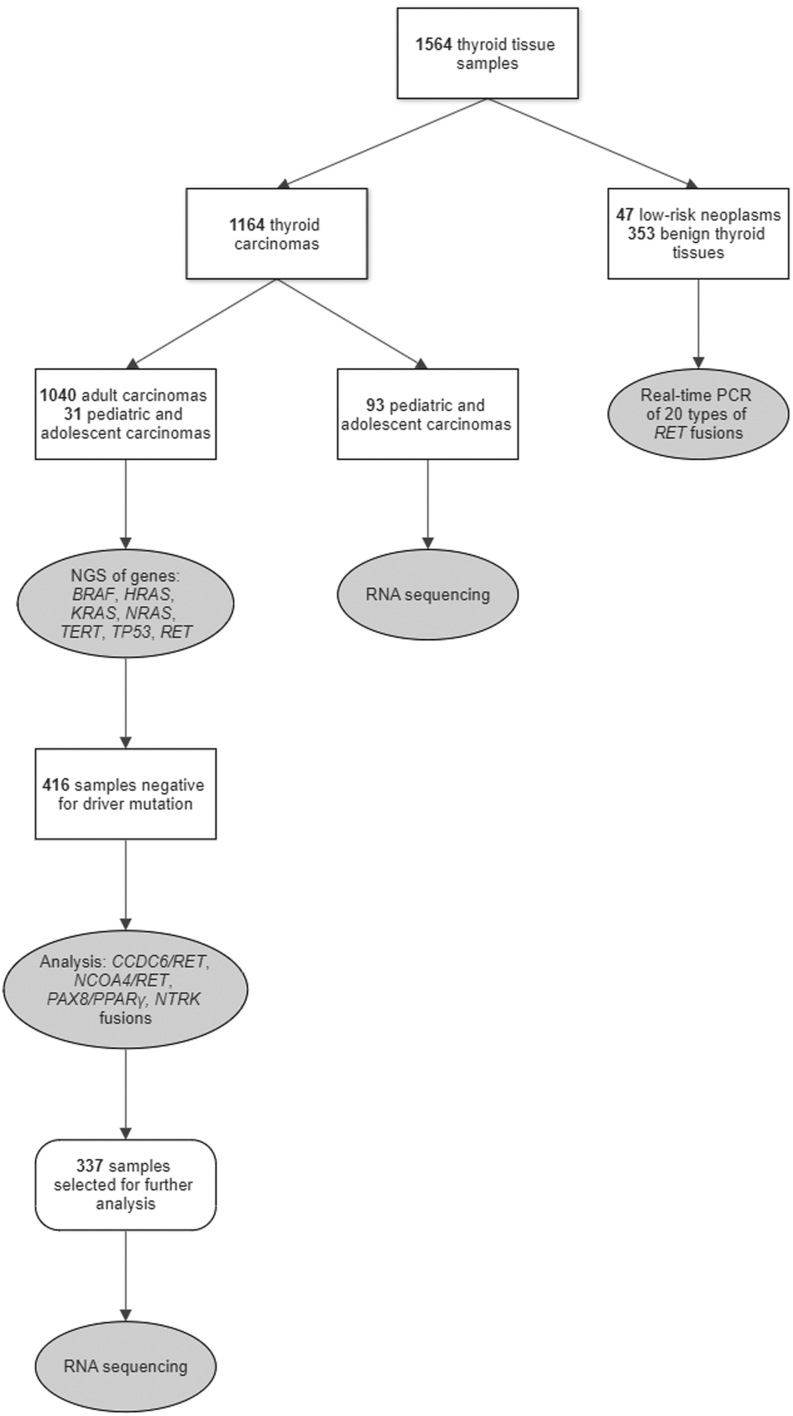
Flowchart illustrates the numbers and types of samples analyzed by different procedures. For pediatric and adolescent carcinomas, RNA sequencing was performed on all samples collected until 2020, and for samples collected between 2020 and 2022, multistage analysis was performed as for carcinomas from adult patients.

The cohort consisted of a total of 1164 thyroid carcinomas (998 PTCs, 98 medullary thyroid carcinomas (MTCs), 24 follicular thyroid carcinomas (FTCs), 16 anaplastic thyroid carcinomas (ATCs), 11 poorly differentiated thyroid carcinomas (PDTCs), and 17 oncocytic carcinomas (OCAs)), 47 low-risk neoplasms (26 follicular tumors of uncertain malignant potential, 18 noninvasive follicular tumors with papillary-like nuclear features, three hyalinizing trabecular tumors), and 353 benign thyroid tissues (adenomas, hyperplastic nodules, chronic lymphocytic thyroiditis (CLT)) (Fig. 1). Thyroid tissue samples were collected from three Prague hospitals: the Motol University Hospital (between years 2003 and 2022), the University Hospital Kralovske Vinohrady (between years 2016 and 2022), and the Military University Hospital (between years 2019 and 2022). One thousand four hundred eighty-one samples were fresh frozen thyroid tissues and 83 samples were formalin-fixed, paraffin-embedded (FFPE) thyroid tissues. Informed consent was obtained from all patients or parents/legal guardians of pediatric patients. All molecular genetic analyses were performed in the laboratory of the Institute of Endocrinology (Department of Molecular Endocrinology). The study was approved by the Ethics Committee of the Institute of Endocrinology in Prague (EK-EÚ/F192/08062020).

### Nucleic acid extraction

DNA and RNA from fresh frozen and FFPE tissues were extracted using the AllPrep DNA/RNA/miRNA Universal Kit and AllPrep DNA/RNA FFPE Kit (Qiagen), respectively and the QIAcube Connect automatic isolator (Qiagen). Quantitative and qualitative analysis of all samples was performed using a fluorometer (Qubit 2.0, Invitrogen) and a spectrophotometer (QIAxpert, Qiagen). Nucleic acid from 11 FFPE samples (five PTCs, three MTCs, one FTC, one OCA, one benign thyroid tissue) was insufficient in quality or quantity for further analyses; thus, these samples were excluded from the study.

### *RET* fusion gene analysis of pediatric samples

The majority of pediatric PTC samples (*n* = 93) was analyzed for the presence of *RET* fusion genes using the QIASeq Targeted RNAscan panel (Qiagen) or FusionPlex Comprehensive Thyroid and Lung (CTL) panel (ArcherDx, Boulder, CO, USA) as reported in our previous study (Pekova *et al.* 2020). Pediatric samples (*n* = 31) collected between 2020 and 2022 were analyzed using the same procedure as adult samples described below (Fig. 1).

### Analysis of point mutations

Adult and newly collected pediatric thyroid cancer samples were first analyzed for point mutations in the following genes: *BRAF* (exon 15), *HRAS* (exons 2 and 3), *KRAS* (exons 2 and 3), *NRAS* (exons 2 and 3), *TERT* (promoter), *TP53* (exons 4, 5, 6, 7, 8, and 9), and *RET* (exons 8, 10, 11, 13, 14, 15, and 16) (Fig. 1). Libraries were prepared using the Nextera XT DNA Library Prep Kit (Illumina), quantified using a fluorometer, and sequenced on the MiSeq (Illumina). *TERT* promoter mutations C228T and C250T were analyzed by capillary sequencing on the CEQ 8000 instrument (Beckman Coulter, Brea, CA, USA) as previously described (Pekova *et al.* 2019) or using the PNAClamp TERT Mutation Detection Kit (Panagene) and real-time PCR using the Light Cycler® 480 (Roche).

### Analysis of selected fusion genes

Carcinoma samples that were negative for a driver point mutation in the *BRAF*, *HRAS*, *KRAS*, *NRAS*,or *RET* genes were further analyzed for the *CCDC6/RET*, *NCOA4/RET*, *PAX8/PPARγ*, and *NTRK* fusions as described in our previous study (Pekova *et al.* 2021) (Fig. 1). This selective analysis approach was chosen because these driver mutations are typically mutually exclusive with other driver mutations (Agrawal *et al.* 2014, Pekova *et al.* 2020, Parimi *et al.* 2023).

### RNA sequencing

RNA sequencing was performed on a total of 337 carcinoma samples that were negative for driver mutations in genes *BRAF*, *HRAS*, *KRAS*, *NRAS*, and *RET* or fusion genes *CCDC6/RET*, *NCOA4/RET*, *PAX8/PPARγ,* and *NTRK* (Fig. 1).

Libraries were prepared by the FusionPlex CTL panel (ArcherDx) using Anchored Multiplex PCR technology, which allowed detection of novel fusion genes. Briefly, total RNA (250 ng) was reverse-transcribed into cDNA using random primers. The cDNA ends were modified to allow ligation of Archer molecular barcode adapters (including i5 indexes). Two rounds of PCR followed. During the second round PCR, i7 indexes were added. Purified libraries were quantified and pooled to equimolar concentrations. The resulting libraries were subjected to paired-end sequencing on the MiSeq (Illumina). Bioinformatic analysis was performed using the Archer Analysis software version 6.2.7 (ArcherDx). For a gene fusion to be considered valid, the breakpoint had to span at least five high-quality unique reads and at least three reads had to have a unique start site.

### Real-time PCR analysis

All *RET* fusion genes identified by the FusionPlex CTL panel were verified by real-time PCR analysis. Total RNA (2 µg) was reverse transcribed to cDNA using random primers, dNTPs, RNase inhibitor and AMV reverse transcriptase (Promega) as described in our previous study (Pekova *et al.* 2020). The cDNA was diluted five times and amplified using the TaqMan Fast Advanced Master Mix (Applied Biosystems) and gene-specific primers and hydrolysis probes that were synthesized based on our design (Supplementary Table 1, see section on supplementary materials given at the end of this article). The *ACTB* gene was used as the reference gene. Each experiment included a positive control and a negative control where RNase-free water was used instead of template cDNA. Real-time PCR was performed as follows on the Light Cycler® 480 (Roche): 50°C for 2 min, 95°C for 20 s followed by 40 cycles of 95°C for 3 s and 60°C for 30 s.

A total of 400 low-risk neoplasms and benign thyroid tissue samples were screened for all 20 types of *RET* fusion genes identified in carcinoma samples in this study (Fig. 1). Testing of the samples was performed using real-time PCR analysis as described above.

### Statistical analysis

Categorical data are summarized as *n* (%). Normally distributed data are summarized as the mean (standard deviation) and non-normally distributed data as the median (range). Categorical variables were compared using the Fisher’s exact test or Pearson’s chi-squared test, and continuous variables were compared using the *t*-test. Statistical analyses were performed using the Simple Interactive Statistical Analysis and the GraphPad tools (GraphPad Software). A *P* < 0.05 was considered statistically significant.

## Results

### Molecular characterization

*RET* fusion genes were found only in PTCs. No *RET* fusion genes were identified in other types of thyroid carcinomas, MTCs, FTCs, ATCs, PDTCs, OCAs, or in any low-risk neoplasms or benign thyroid lesions. *RET* fusions were detected in 113/993 (11.4%) patients with PTC.

**Figure 2:**
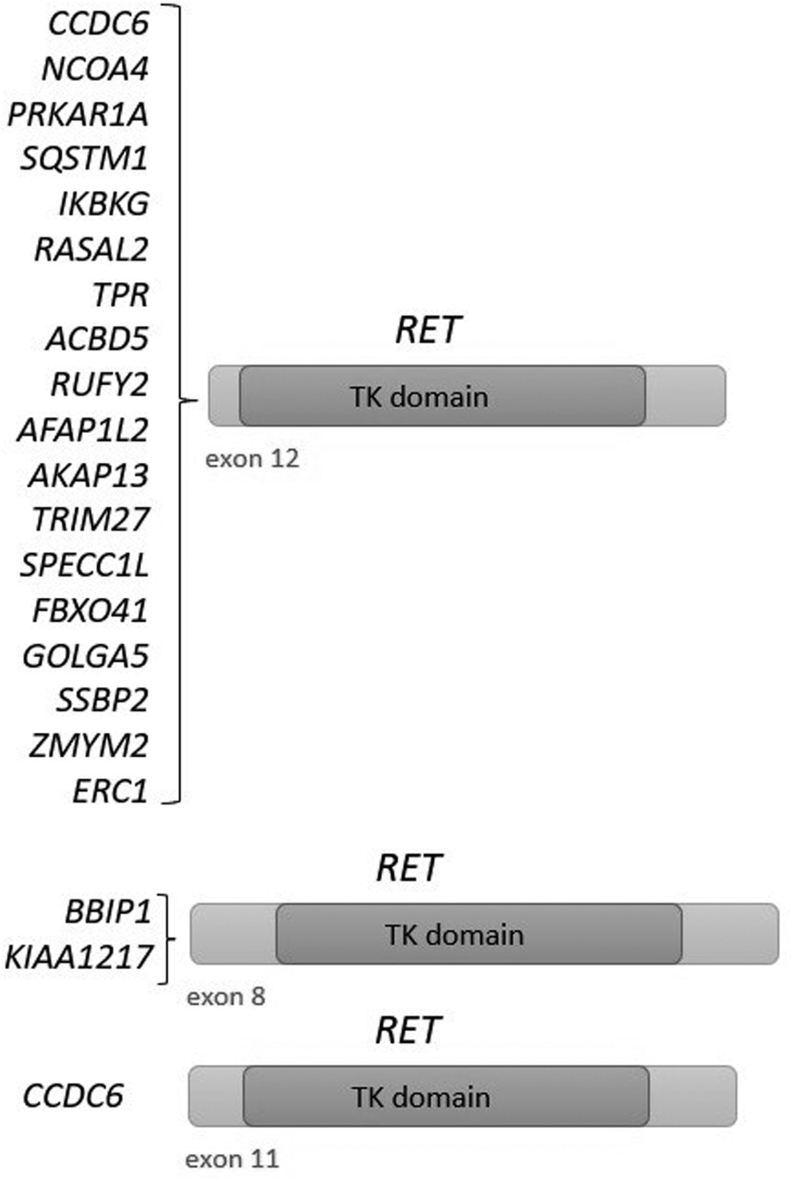
Overview of identified *RET* fusion genes. Partner genes were most frequently fused to exon 12 of the *RET* gene, but in rare cases also to exon 8 and exon 11 of the *RET* gene. TK, tyrosine kinase.

A total of 20 different types of *RET* fusion genes were identified (Fig. 2). Partner genes were as follows: *ACBD5*, *AFAP1L2*, *AKAP13*,* BBIP1*, *CCDC6*, *ERC1*, *FBXO41*, *GOLGA5*, *IKBKG*, *KIAA1217, NCOA4*, *PRKAR1A*, *RASAL2*, *RUFY2*, *SPECC1L*, *SQSTM1*, *SSBP2*, *TPR*, *TRIM27*, *ZMYM2*. The *CCDC6* partner gene was detected predominantly in 67 (59.3%) cases, followed by the *NCOA4* gene, which was found in 25 (22.1%) cases. The partner genes *RUFY2*, *AFAP1L2*, and *PRKAR1A* were also recurrent, while the others were detected in only one case. The *FBXO41/RET, SSBP2/RET,*and *ZMYM2/RET* fusion genes were novel, all of them were in-frame. In two cases, two different coexisting *RET* fusions were identified in each sample (*ACBD5/RET* + *BBIP1/RET*; *SPECC1L/RET* + *FBXO41/RET*). In some cases of *CCDC6/RET* and *NCOA4/RET* fusion genes, different isoforms were revealed (Supplementary Table 2).

The *TERT* promoter mutation was detected along with the *CCDC6/RET* fusion in two male patients. In two multifocal cases, the *CCDC6/RET* fusion gene was detected in one nodule and the *BRAF* V600E mutation in the other. In one unique case of a female, different multiple mutations were found in her three thyroid nodules: *CCDC6/RET* + *NRAS* Q61R in the first nodule, *CCDC6/RET* + *KRAS* G13D in the second nodule, and *NRAS* Q61R + *PTEN* Q245* in the third nodule. Her lymph node metastases (LNM) were positive only for the *CCDC6/RET* fusion gene.

### Clinicopathological characteristics

**Figure 3:**
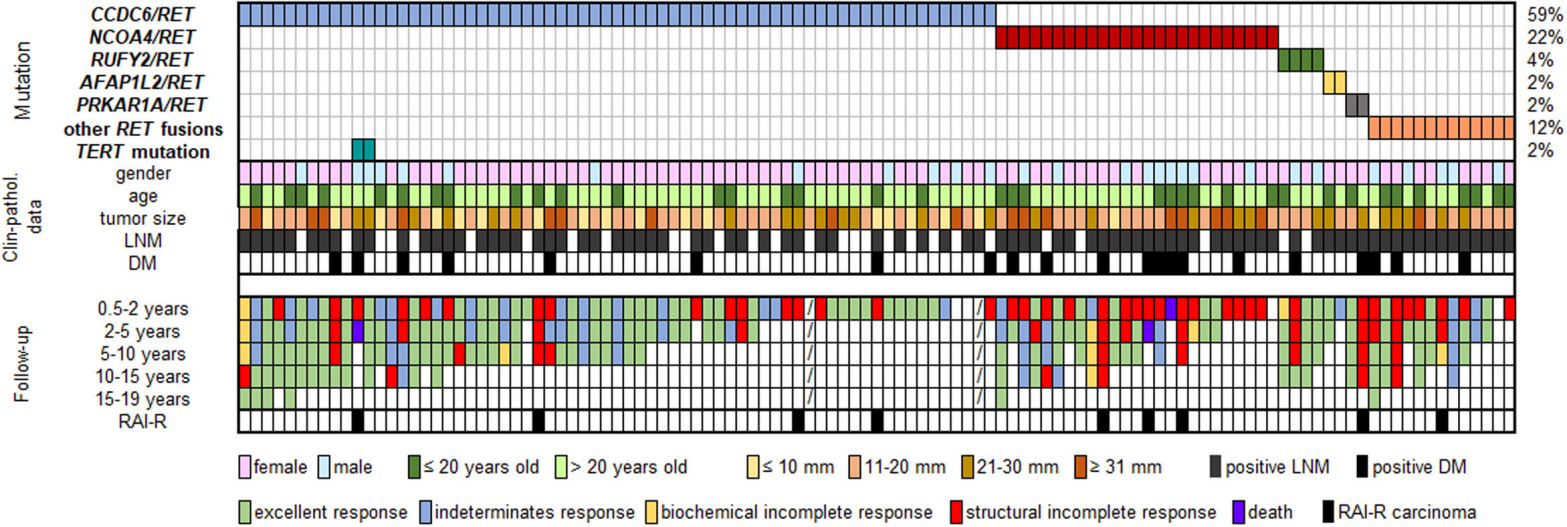
Tile plot of patients with *RET* fusion-positive carcinoma, their clinicopathological and follow-up data. Clinical and pathological data such as gender, age at diagnosis, tumor size, lymph node metastases, and distant metastases are shown. Follow-up data display patient´s response to treatment and cases of radioiodine-refractory carcinomas. LNM, lymph node metastases; DM, distant metastases; RAI-R, radioactive iodine-refractory.

Clinicopathological data of patients with *RET* fusion-positive carcinoma are summarized in Table 1 and Fig. 3. The cohort consisted of 113 patients, 85 (75.2%) females and 28 (24.8%) males, with the mean age at diagnosis of 32.6 ± 17.4 years. Four patients had malignant disease (invasive adenocarcinoma of the lung, prostate cancer, dermatofibrosarcoma protuberans, and non-Hodgkin lymphoma) before the diagnosis of PTC. The mean tumor size was 21.8 ± 12.6 mm. Microcarcinoma (≤10 mm) had 16% of patients. In one case, only PTC metastases in lymph nodes were found in a patient without an identified primary thyroid tumor. A combination of papillary and follicular growth patterns on histology was predominant, found in 37 cases, and in a further nine cases the specimens also had a component of a solid growth pattern. Less frequent histological subtypes were diffuse sclerosing variant identified in five cases and solid and clear cell variant both in two cases. Multifocality was found in 48.2% of cases and 66.7% of these cases had numerous, in some cases up to hundreds, intrathyroidal microcarcinomas. Tumors usually showed infiltrative growth in the thyroid parenchyma and were unencapsulated. The majority (75.2%) of patients had LNM, of which 21.2% were located only in the central compartment and 78.8% in the lateral compartment, and 18.6% had evidence of distant metastases (DM), which in all cases affected the lungs, additionally in two cases the bones. The frequency of LNM and DM in association with primary tumor size is shown in Table 2. Tumor size was divided into four categories: ≤ 10 mm, 11–20 mm, 21–30 mm, and ≥31 mm, and the data were analyzed separately for adult and pediatric patients. LNM and DM also occurred frequently in patients with microcarcinoma.

**Table 1:** Clinical and pathological features of patients harboring *RET* fusion-positive carcinomas.

	All* RET* fusion-positive PTCs (%) *n* = 113	Adult (%) *n* = 76	Pediatric and adolescent (%) *n* = 37	*P*
Patients				
Females/males	85/28	59/17	26/11	0.266
Age at diagnosis (mean ± s.d.)	32.6 ± 17.4	42.2 ± 12.8	13.0 ± 3.8	
Radiation exposure before the diagnosis of PTC	5 (4.4)	5 (6.6)	0	0.132
Malignancy before the diagnosis of PTC	4 (3.5)	4 (5.3)	0	0.199
Tumor size				
Mean ± s.d. (mm)	21.8 ± 12.6	19.2 ± 10.4	26.8 ± 14.8	**0.002**
Microcarcinoma (≤10 mm)	18 (16.0)	14 (18.4)	4 (10.8)	0.226
Histology				
Predominantly papillary growth pattern	23 (20.4)	17 (22.4)	6 (16.2)	0.309
Mixture of papillary and follicular growth pattern	37 (32.7)	23 (30.3)	14 (37.8)	0.275
Mixture of papillary, follicular, and solid growth pattern	9 (8.0)	5 (6.6)	4 (10.8)	0.331
Predominantly follicular growth pattern	24 (21.2)	17 (22.4)	7 (18.9)	0.437
Other	9 (8.0)	5 (6.6)	4 (10.8)	0.331
Unknown	11 (9.7)	9 (11.8)	2 (5.4)	0.234
Pathological characteristics				
Multifocality	54 (48.2)	34/75 (45.3)	20 (54.1)	0.252
Numerous intrathyroidal microcarcinomas	36 (32.4)	20/75 (26.7)	16/36 (44.4)	0.050
Extrathyroidal extension	47 (43.5)	24/71 (33.8)	23 (62.2)	**0.004**
Intravascular invasion	34 (33.3)	19/65 (29.2)	15 (40.5)	0.172
Lymph node metastases	85 (75.2)	52 (68.4)	33 (89.2)	**0.012**
Distant metastases	21 (18.6)	12 (15.8)	9 (24.3)	0.200
Chronic lymphocytic thyroiditis	73 (65.2)	48/75 (64.0)	25 (67.6)	0.439
Frequent psammoma bodies	32 (28.8)	22/75 (29.3)	10/36 (27.8)	0.649

Values highlighted in bold were statistically significant.

PTC, papillary thyroid carcinoma.

**Table 2:** Frequency of LNM and DM in relation to primary tumor size in adult and pediatric and adolescent patients with *RET* fusion-positive carcinoma.

Patients	Metastases	Tumor size			
		≤10 mm	11–20 mm	21–30 mm	≥ 31 mm
Adults	LNM	11 (73%)	23 (62%)	12 (75%)	6 (75%)
	DM	2 (13%)	4 (11%)	4 (25%)	2 (25%)
Pediatric and adolescent	LNM	3 (75%)	9 (75%)	11 (100%)	10 (100%)
	DM	1 (25%)	1 (8%)	3 (27%)	4 (40%)

Of the 113 patients with PTC positive for the *RET* fusion gene, 76/869 (8.7%) were adults and 37/124 (29.8%) were pediatric and adolescent patients aged 5 to 20 years. A comparison of the two cohorts is shown in Table 1. Pediatric patients had a significantly larger mean tumor size compared to adults, 26.8 mm vs 19.2 mm (*P* = 0.002). Other features in which pediatric patients differed significantly from adults were extrathyroidal extension (62.2% vs 33.8%; *P* = 0.004) and LNM (89.2% vs 68.4%; *P* = 0.012).

*NCOA4/RET* fusion-positive carcinomas were compared with carcinomas harboring other *RET* fusions (Table 3). PTCs positive for *NCOA4/RET* were significantly associated with larger tumor size (*P* = 0.021) and more frequently had extrathyroidal extension (*P* = 0.049), in contrast to carcinomas positive for other *RET* fusions, which were significantly associated with a predominant papillary growth pattern (*P* = 0.001) and CLT (*P* = 0.005). Patients with PTC positive for *NCOA4/RET* had more frequent LNM and DM than patients with other *RET* fusions; however, the differences only approached the threshold of statistical significance (*P* = 0.074 and *P* = 0.053, respectively).

**Table 3:** Comparison of clinical and pathological features between *NCOA4/RET* and other *RET* fusion-positive PTCs.

	*NCOA4/RET*-positive PTCs (%)*n* = 25	Other* RET* fusion-positive PTCs (%) *n* = 88	*P*
Patients			
Females/males	16/9	69/19	0.115
Age at diagnosis (mean ± s.d.)	33.6 ± 19.9	32.4 ± 16.6	0.761
Radiation exposure before the diagnosis of PTC	1 (4.0)	4 (4.5)	0.721
Malignancy before the diagnosis of PTC	1 (4.0)	3 (3.4)	0.638
Tumor size			
Mean ± s.d. (mm)	26.8 ± 15.1	20.3 ± 11.3	**0.021**
Microcarcinoma (≤10 mm)	1 (4.0)	17 (19.5)	0.050
Histology			
Predominantly papillary growth pattern	0	23 (26.1)	**0.001**
Mixture of papillary and follicular growth pattern	9 (36.0)	27 (30.7)	0.392
Mixture of papillary, follicular, and solid growth pattern	1 (4.0)	8 (9.1)	0.365
Predominantly follicular growth pattern	8 (32.0)	16 (18.2)	0.114
Other	4 (16.0)	5 (5.7)	0.107
Unknown	3 (12.0)	8 (9.1)	0.456
Pathological characteristics			
Multifocality	15 (60.0)	38/87 (43.7)	0.113
Numerous intrathyroidal microcarcinomas	9/24 (37.5)	26/87 (29.9)	0.317
Extrathyroidal extension	14/23 (60.9)	33/85 (38.4)	**0.049**
Intravascular invasion	10/21 (47.6)	24/81 (29.6)	0.098
Lymph node metastases	22 (88.0)	63 (71.6)	0.074
Distant metastases	8 (32.0)	13 (14.8)	0.053
Chronic lymphocytic thyroiditis	10 (40.0)	62/87 (71.3)	**0.005**
Frequent psammoma bodies	6/24 (25.0)	26/87 (29.9)	0.424

Values highlighted in bold were statistically significant.

PTC, papillary thyroid carcinoma.

Comparison of clinicopathological features between PTCs positive for *RET* fusions and individual groups of PTCs with *NTRK* fusions, *BRAF* V600E and *RAS* mutations was performed (Table 4). Only patients with available clinicopathological data were included. Patients with *RET* fusion-positive PTCs were significantly younger than patients with *BRAF* V600E and *RAS*-mutated carcinomas (*P* < 0.001) and had significantly larger tumor than patients with the *BRAF* V600E-mutated carcinomas (*P* < 0.001). In *RET* fusion-positive PTCs, features associated with higher tumor aggressiveness were detected more often than in PTCs positive for other mutations. LNM and DM were significantly more frequent in patients with *RET* fusions than in patients with other mutations. Multifocality, extrathyroidal extension, and intravascular invasion were significantly more common in *RET* fusion-positive PTCs than in *BRAF* V600E and *RAS*-mutated PTCs.

**Table 4:** Comparison of clinicopathological features between PTCs positive for *RET* and *NTRK* fusions,* BRAF* V600E and *RAS* mutations.

	RET fusions (%) *n* = 113	NTRK fusions (%) *n* = 57	*P* (*RET* fusions × *NTRK* fusions)	BRAF *V600E* (%) *n* = 413	*P* (*RET* fusions × *BRAF V600E *)	RAS (%) *n* = 61	*P* (*RET* fusions × *RAS*)
Patients							
Females/males	85/28	45/12	0.367	323/90	0.289	50/11	0.205
Age at diagnosis (mean ± s.d.)	32.6 ± 17.4	32.1 ± 15.2	0.854	47.1 ± 16.7	**<0.001**	45.1 ± 17.3	**<0.001**
Radiation exposure before the diagnosis of carcinoma	5 (4.4)	1 (1.8)	0.343	NA	/	NA	/
Malignancy before the diagnosis of carcinoma	4 (3.5)	1 (1.8)	0.455	32 (7.7)	0.081	4 (6.6)	0.495
Tumor size							
Mean ± s.d. (mm)	21.8 ± 12.6	20.2 ± 11.3	0.420	17.6 ± 10.2	**<0.001**	20.0 ± 18.5	0.449
Microcarcinoma (≤10 mm)	18 (16.0)	8 (14.0)	0.468	99 (24.0)	**0.042**	17/60 (28.3)	**0.043**
Histology (*n*)	102	57		375		59	
Predominantly papillary growth pattern	23 (22.5)	8 (14.0)	0.137	193 (51.5)	**<0.001**	8 (13.6)	0.117
Mixture of papillary and follicular growth pattern	37 (36.3)	18 (31.6)	0.338	114 (30.4)	0.258	6 (10.2)	**<0.001**
Mixture of papillary, follicular, and solid growth pattern	9 (8.8)	0	**0.016**	3 (0.8)	**<0.001**	0	**0.013**
Predominantly follicular growth pattern	24 (23.5)	28 (49.1)	**<0.001**	41 (10.9)	**0.001**	42 (71.2)	**< 0.001**
Other	9 (8.8)	3 (5.3)	0.316	24 (6.4)	0.256	3 (5.1)	0.295
Pathological characteristics							
Multifocality	54/112 (48.2)	25 (43.9)	0.355	146/392 (37.2)	**0.036**	15/57 (26.3)	**0.005**
Extrathyroidal extension	47/108 (43.5)	22 (38.6)	0.330	125/408 (30.6)	**0.012**	6 (9.8)	**<0.001**
Intravascular invasion	34/102 (33.3)	11/49 (22.4)	0.118	51/376 (13.6)	**<0.001**	8/58 (13.8)	**0.005**
Lymph node metastases	85 (75.2)	30 (52.6)	**0.003**	142/388 (36.6)	**<0.001**	6/53 (11.3)	**<0.001**
Distant metastases	21 (18.6)	3 (5.3)	**0.013**	10/371 (2.7)	**< 0.001**	3/52 (5.8)	**0.022**
Chronic lymphocytic thyroiditis	73/112 (65.2)	38/56 (67.9)	0.434	NA	/	NA	/
Frequent psammoma bodies	32/111 (28.8)	6/56 (10.7)	**0.006**	NA	**/**	NA	**/**

Values highlighted in bold were statistically significant.

NA, not available.

### Treatment and follow-up

Total thyroidectomy (TTE) was performed in all patients except one who underwent hemithyroidectomy (HTE) and whose follow-up data were not available. Two patients underwent first HTE followed by completion to TTE. Lymph node dissection for suspected LNM was performed in 88 (77.9%) patients. Twenty (17.7%) patients had reoperation, including 19 patients for LNMand one patient for elimination of thyroid remnants.

One hundred and four (92%) patients received radioactive iodine (RAI) treatment. Six patients (five adults, one pediatric patient) were not indicated for RAI therapy due to small tumor size (≤12 mm) and the absence of metastases. One pediatric patient died due to late surgical treatment and massive lung metastases before RAI treatment. Follow-up data of two patients were not available. Approximately half of the patients (55% of adult and 46% of pediatric patients) received one therapeutic dose of RAI, 20% of adults and 19% of pediatric patients received two doses, and 18% of adult and 30% of pediatric patients received three or more doses. The median activity administrated per therapeutic session was 120 mCi (range: 50–200 mCi) equally in adult and pediatric patients. In addition to RAI therapy, two patients with RAI-refractory PTC were treated with radiotherapy and one of them also with chemotherapy. One patient was on targeted treatment with the RET inhibitor selpercatinib. The patient’s tumor infiltrated the respiratory tract, the lesion was inoperable and the patient also had lung metastases. The patient responded well to treatment; he had a partial remission.

Median follow-up was 75 months (range: 4–228 months). Response to treatment was determined based on the definitions and criteria in the 2015 American Thyroid Association Guidelines as an excellent response (ER), an indeterminate response (IR), a biochemical incomplete response (BIR), a structural incomplete response (SIR) (Haugen *et al.* 2015) or death. Response to treatment was assessed in the following time periods after surgery: 0.5–2 years, 2–5 years, 5–10 years, 10–15 years, and 15–19 years. Detailed follow-up data describing the course of treatment for individual patients are shown in Fig. 3. Follow-up data were unknown for two patients and four patients were not evaluated due to short-term follow-up (less than 6 months after surgery).

During the first 2 years after surgery, the number of patients who had ER and SIR to treatment was similar. Later, the patient’s response to treatment improved, although only slightly from the fifth year after surgery. Forty-one (38.3%) patients had ER to treatment during the entire follow-up. Two adult patients (2.4%) had a ‘true recurrence’, a relapse after achieving structural and biochemical disease-free status. LNM were identified in the first case 4 years after surgery and in the second case 7 years after surgery. Four adult patients (4.8%) had SIR after IR/BIR. In three cases, the evidence of metastases (2× LNM, 1× DM) was found around 10 years after surgery and in one case of DM 5 years after surgery. Twelve patients (14.6%, an equal proportion of pediatric and adult patients) had ongoing SIR more than 2 years after surgery. Three patients (2.7%) died and all of them due to PTC. All were males, two adults and one pediatric patient. Two patients had PTC positive for *NCOA4/RET* and one patient for *CCDC6/RET* along with the *TERT* C228T mutation. The 2-, 5-, 10-year disease-specific survival rates were 99%, 96%, and 95%, respectively, for *RET* fusion-positive patients.

According to published definitions, nine (8.7%) patients had RAI-refractory PTC (Tuttle *et al.* 2019). Among these patients were both adults and children, and most were males. Six carcinomas were RAI-refractory from the start of RAI treatment, and in three cases the refractoriness developed over time. RAI-refractory LNM were in seven cases, of which two patients also had RAI-refractory lung metastases and two patients had only RAI-refractory lung metastases.

**Figure 4:**
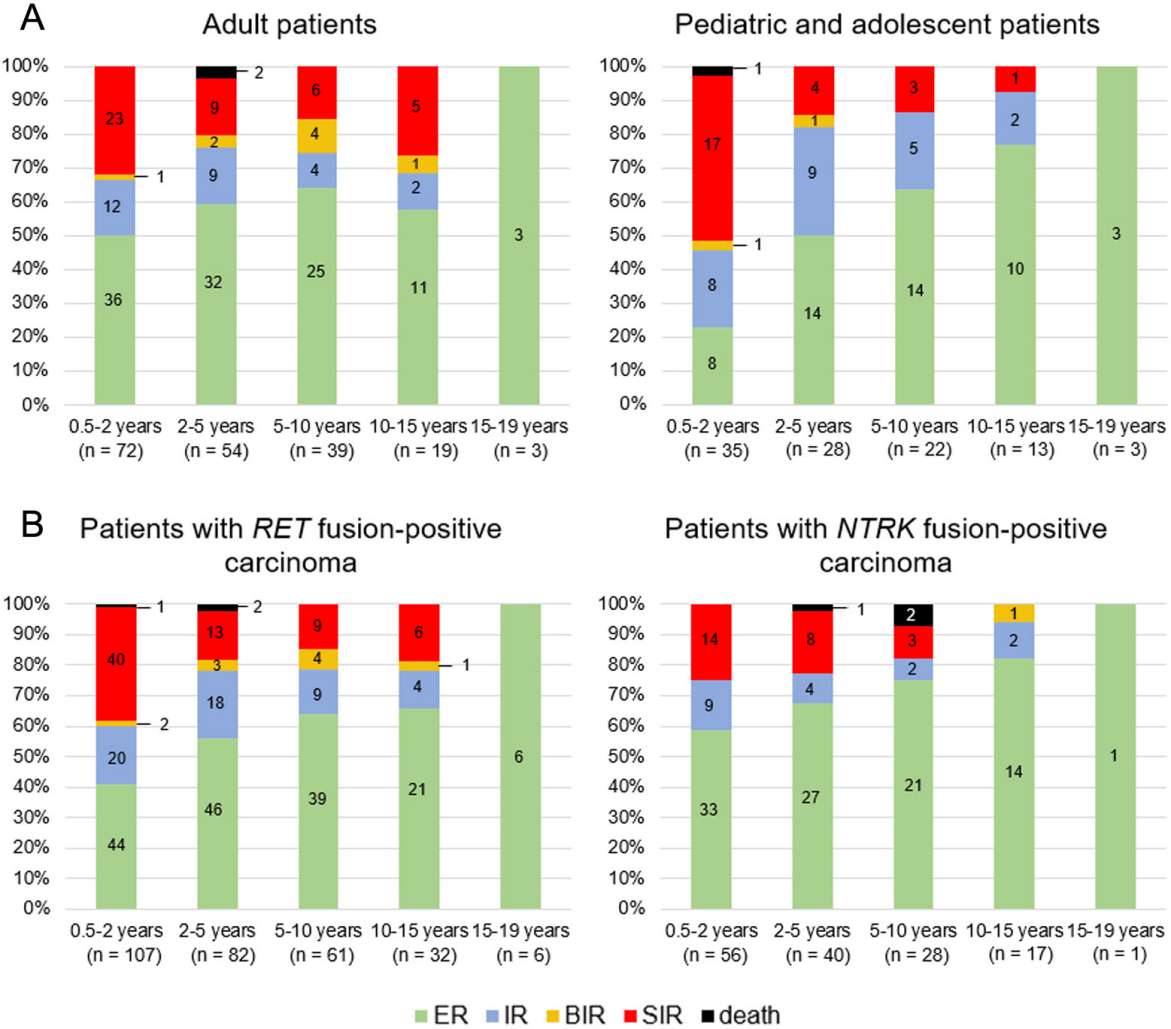
Graphical representation of the responses to treatment during follow-up periods (A) in adult and pediatric and adolescent patients with *RET* fusion-positive carcinomas (B) in all *RET* fusion-positive carcinomas and *NTRK* fusion-positive carcinomas. The numbers in the columns indicate the number of patients. ER, excellent response; IR, indeterminate response; BIR, biochemical incomplete response; SIR, structural incomplete response.

Response to treatment was compared between pediatric and adult patients (Fig. 4A). In the first 2 years after surgery, a significantly lower number of pediatric patients than adult patients had an ER to treatment (*P* = 0.006), probably due to more advanced disease in pediatric patients. Later, improvement was demonstrable in both cohorts and response to treatment became similar in both groups. Ten years after surgery, more pediatric than adult patients had an ER to treatment due to recurrences in the adult group.

Response to treatment was also compared between *RET* fusion and *NTRK* fusion-positive patients (Fig. 4B). Data on *NTRK* fusion-positive patients were used from our previous study (Pekova *et al.* 2021). In the first 2 years after surgery, a significantly lower percentage of *RET* fusion-positive patients than *NTRK* fusion-positive patients had an ER to treatment (*P* = 0.023). Ten years after surgery, no *NTRK* fusion-positive patient had SIR to treatment, in contrast to 19% of *RET* fusion-positive patients.

## Discussion

Thyroid cancer is associated with a broad range of different mutations, including *RET* fusion genes. Previously, the frequency of *RET* fusion genes included only the most common *CCDC6/RET* and *NCOA4/RET* fusions, and other *RET* fusions were rarely tested. To our knowledge, this is one of the most comprehensive studies of a *RET* fusion-positive cohort of thyroid carcinomas that has been analyzed and correlated with clinicopathological and follow-up data.

The frequency of *RET* fusion genes in our adult PTC cohort was 8.7%, which is in the range of 4.3–9.1% as previously reported (Agrawal *et al.* 2014, Lu *et al.* 2017, Liang *et al.* 2018, Yang *et al.* 2021, Shi *et al.* 2022, Ullmann *et al.* 2022, Wang *et al.* 2022, Parimi *et al.* 2023). Pediatric and adolescent PTCs harbored *RET* rearrangements more than three times more frequently than adult PTCs, in 29.8% of cases. This result is within the range of 10.4–57.7% previously published results (Ricarte-Filho *et al.* 2013, Galuppini *et al.* 2019, Pekova *et al.* 2020, Macerola *et al.* 2021, Rogounovitch *et al.* 2021, Franco *et al.* 2022, Satapathy & Bal 2022). The variability is likely due to the range of methods used, the size and composition of cohorts and geographic location.

Next-generation sequencing (NGS) is currently the most reliable method for detection of *RET* fusion genes. The use of a comprehensive panel for the detection of fusion genes should be the gold standard, especially in pediatric thyroid carcinomas, in which the occurrence of fusions prevails. A more cost-effective approach using mutational exclusivity of *RET* fusions with other driver mutations could be applied in adult thyroid carcinomas. First, detection of point driver mutations in the *BRAF* and *RAS* genes would be performed. Negative samples would then be analyzed for the most common *CCDC6/RET* and *NCOA4/RET* fusions, representing approx. Eighty percent of all *RET* fusions by real-time PCR. Negative samples would undergo NGS testing, which would also allow the detection of novel fusion partner genes. To date, at least 50 *RET* partner genes have been described in thyroid cancer, and the number has been rising steeply in recent years (Agrawal *et al.* 2014, Grubbs *et al.* 2015, Yakushina *et al.* 2017, Pekova *et al.* 2020, Lee *et al.* 2021, Rogounovitch *et al.* 2021, Yang *et al.* 2021, Franco *et al.* 2022, Wang *et al.* 2022).

In this study, *RET* fusion genes were detected only in PTCs. A few rare cases of *RET* rearrangements in PDTCs, in eight ATCs, and in two MTCs have been described in the literature (Grubbs *et al.* 2015, Landa *et al.* 2016, Yang *et al.* 2021, Parimi *et al.* 2023). There are also studies in which *RET* fusions have been found in benign samples, particularly in Hashimoto’s thyroiditis (Wirtschafter *et al.* 1997, Sheils *et al.* 2000, Elisei *et al.* 2001). Most of these studies were performed around year 2000, when the methods used had substantial technical limitations leading to a high risk of false-positive results (Nikiforov 2006). Although no recent case of *RET* fusion in a benign thyroid sample has been reported, their presence in these tissues has been questioned (Nikiforova *et al.* 2002), the occurrence of *RET* fusions in Hashimoto’s thyroiditis has been reported in recent review articles (Rangel-Pozzo *et al.* 2020, Nacchio *et al.* 2022). In our study, no *RET* fusion was detected in any of the benign samples. Based on these results we conclude that the identification of an oncogenic *RET* fusion is associated with a 100% probability of malignancy.

Coexistence of CLT with *RET* fusion-positive PTC was found in 65.2% of our cases, similarly in pediatric and adult patients. The frequency of this coexistence is higher than the range of 15–40% reported in the literature for PTC cohorts with unknown genetic background (Kim *et al.* 2009, Babli *et al.* 2018, Lee *et al.* 2020). In another study, the authors found a negative correlation between the coexistence of CLT with PTC and the *BRAF* mutation (Lee *et al.* 2020). In addition, the presence of numerous psammoma bodies was found in our study. The association between them and *RET* fusions has been previously described (Adeniran *et al.* 2006). Thus, the presence of coexisting CLT as well as numerous psammoma bodies could indicate a higher probability of *RET* fusion gene in PTC.

Other examined clinicopathological features that were abundantly identified in *RET* fusion-positive PTCs were multifocality, extrathyroidal extension, intravascular invasion, LNM, and DM. *RET* fusion-positive carcinomas were associated with aggressive and invasive behavior. This correlation was also noticed by the authors of studies on small *RET* fusion-positive cohorts (Franco *et al.* 2022, Ullmann *et al.* 2022, Wang *et al.* 2022).

Multifocality was detected in 48.2% of *RET* fusion-positive carcinomas, which was significantly higher than in our *BRAF* V600E and *RAS*-mutated PTCs. In another study, multifocality was even found in 86% of *RET* fusion-positive PTCs (Ullmann *et al.* 2022). Most of our patients with multifocal PTC had numerous millimeter-sized intrathyroidal microcarcinomas. The frequency of these cases may have been even higher due to several cases, in which the lobes were filled with carcinomas and may have been formed by the union of multiple foci. The sensitivity of ultrasound for these clinically occult lesions is low. Thus, TTE should be considered in the case of preoperative detection of an oncogenic *RET* fusion gene in a thyroid nodule.

*RET*-fusion positive patients had a significantly higher incidence of LNM and DM compared to patients with *NTRK* fusions, *BRAF* V600E or *RAS* mutations. In *RET*-fusion positive patients, LNM were identified in 68.4% of adult and 89.2% of pediatric patients. Other studies reached similar results, in which 91–94% of pediatric patients with *RET* fusion-positive PTCs had LNM (Lee *et al.* 2021, Franco *et al.* 2022). In our cohort, the majority of patients had evident multiple LNM in the lateral compartment. The correlation of *RET* fusion-positive PTCs with LNM in the lateral compartment was also noted by the authors of other studies (Rogounovitch *et al.* 2021, Wang *et al.* 2022). Therefore, sonographic evaluation of the lymph nodes in the central and lateral compartments should be performed conscientiously due to the high probability of LNM regardless of tumor size.

Differences were also observed within the *RET* fusion gene cohort. PTCs positive for *NCOA4/RET* had significantly larger tumors and more frequent extrathyroidal extension. In addition, PTCs positive for the *NCOA4/RET* fusion had a tendency to be associated with more LNM and DM, although this finding was not statistically significant. The correlation between the *NCOA4/RET* fusion and higher aggressiveness has been mentioned in other studies (Galuppini *et al.* 2019, Rogounovitch *et al.* 2021).

Follow-up data on patients with *RET* fusion-positive PTC are very scarce in the literature. In one study, 50% of *RET* fusion-positive pediatric patients achieved remission one year after surgery (Franco *et al.* 2022), a high percentage considering that 94% of patients had LNM and 35% had DM. In another study, 24% and 19% of *RET* fusion-positive pediatric patients had ER and IR/BIR to treatment, respectively, throughout follow-up. SIR to treatment had 57% of patients, of whom 14% achieved ER or biochemical disease without structural evidence of disease at last follow-up (Lee *et al.* 2021). Compared to our study, a similar proportion of patients had SIR to treatment, but significantly more of our pediatric patients achieved clinical improvement. An explanation could be more advanced disease of their patients, 38% vs our 25% patients with DM, and the shorter follow-up in their study (Lee *et al.* 2021). Follow-up data on adult *RET* fusion-positive patients are even scarcer. In one study, none of the *RET* fusion-positive patients relapsed and 2/14 (14%) patients had persistent disease (Ullmann *et al.* 2022).

Only 8.7% of our patients had RAI-refractory carcinoma, which is within the reported range of 5–15% for differentiated thyroid carcinomas (Worden 2014). Only one patient in our cohort had treatment with selpercatinib. Promising results of this type of treatment from other studies have already been published (Lee *et al.* 2021).

Despite more aggressive disease of the *RET*-fusion positive cohort, the 2.4% ‘true recurrence’, 2.7% cancer-specific mortality, 96% and 95% survival at 5 and 10 years were comparable to data (approximately 1% ‘true recurrence’, 2–2.5% mortality, 99% and 95% survival at 5 and 10 years) for PTC cohorts with unknown genetic background (Sciuto *et al.* 2009, Ito *et al.* 2018). It is important to note that the majority of patients in our cohort had intensive treatment, relatively radical surgery and many patients underwent repeated RAI therapy. In addition, the patients’ condition was regularly monitored over a long period of time, which is essential for a good outcome, as recurrence can occur many years after surgery. It is questionable what the response to treatment would have been with a milder treatment (e.g. lobectomy and no RAI treatment), whether or not the recurrence and mortality rates would have been significantly higher.

A limitation of the study was that no functional analyses of the novel *RET* fusions were performed. However, the portion encoding the RET kinase domain was retained intact in all novel *RET* fusions. In addition, all partner genes contained domains enabling dimerization required for oncogenic activation, the *ZMYM2* gene encodes a zing finger protein, the *SSBP2* gene encodes a tumor suppressor protein containing a LisH domain and the *FBOX41* gene encodes an F-box protein containing leucine-rich repeats.

In summary, *RET* fusion genes were detected in 11.4% of PTC patients; in 8.7% of adult and 29.8% of pediatric patients with PTC. *RET* fusion-positive carcinomas were associated with aggressive disease, including frequent LNM and DM, especially in cases of *NCOA4/RET*-positive carcinomas, even in early-stage disease, and with increased rates in pediatric and adolescent patients. The presence of the *RET* fusion gene could be indicated by the occurrence of CLT and numerous psammoma bodies. In case of preoperative detection of the *RET* fusion gene, malignancy should be expected, and a high probability of metastases should be anticipated. Recurrences were rare and occurred only in adult patients. Although most patients did not achieve remission soon after surgery and half of the pediatric patients had structural disease in the first 2 years after surgery, the prognosis was favorable from a long-term perspective, but with regular follow-up.

## Supplementary Materials

Supplementary Table S1. Sequences of primers and hydrolysis probes designed for the ACTB gene and the RET fusion genes detection using Real-Time PCR

Supplementary Table S2. Isoforms and transcripts of genes involved in RET rearrangements

## Declaration of interest

The authors declare that there is no conflict of interest that could be perceived as prejudicing the impartiality of the research reported.

## Funding

This work was supported by the Ministry of Health http://dx.doi.org/10.13039/100009647 of the Czech Republic AZV (NU21-01-00448) and MH CZ-DRO (Institute of Endocrinology, 00023761) grants.
